# Exposure to salinity induces oxidative damage and changes in the expression of genes related to appetite regulation in Nile tilapia (*Oreochromis niloticus*)

**DOI:** 10.3389/fgene.2022.948228

**Published:** 2022-09-08

**Authors:** Amanda W. S. Martins, Eduardo N. Dellagostin, Eduardo B. Blödorn, Tony Leandro R. Silveira, Luis A. Sampaio, Eliza R. Komninou, Antonio S. Varela Junior, Carine D. Corcini, Leandro S. Nunes, Mariana H. Remião, Gilberto L. Collares, William B. Domingues, Vinicius F. Campos

**Affiliations:** ^1^ Laboratório de Genômica Estrutural, Programa de Pós-Graduação em Biotecnologia, Centro de Desenvolvimento Tecnológico, Universidade Federal de Pelotas, Pelotas, RS, Brazil; ^2^ Instituto de Ciências Biológicas, Universidade Federal do Rio Grande, Rio Grande, RS, Brazil; ^3^ Laboratório de Piscicultura Estuarina e Marinha, Programa de Pós-graduação em Aquicultura, Instituto de Oceanografia, Universidade Federal do Rio Grande, Rio Grande, RS, Brazil; ^4^ Laboratório de Reprodução Animal, Programa de Pós-Graduação em Biologia de Ambientes Aquáticos Continentais, Instituto de Ciências Biológicas, Universidade Federal do Rio Grande, Rio Grande, RS, Brazil; ^5^ ReproPel, Programa de Pós-Graduação em Veterinária, Faculdade de Veterinária, Universidade Federal de Pelotas, Pelotas, RS, Brazil; ^6^ Agência de Desenvolvimento da Bacia da Lagoa Mirim, Universidade Federal de Pelotas, Pelotas, RS, Brazil

**Keywords:** CART, PYY, NPY, CCK, gene expression, food intake

## Abstract

Variations in water salinity and other extrinsic factors have been shown to induce changes in feeding rhythms and growth in fish. However, it is unknown whether appetite-related hormones mediate these changes in Nile tilapia (*Oreochromis niloticus*)*,* an important species for aquaculture in several countries. This study aimed to evaluate the expression of genes responsible for appetite regulation and genes related to metabolic and physiological changes in tilapia exposed to different salinities. Moreover, the study proposed to sequence and to characterize the *cart, cck*, and *pyy* genes, and to quantify their expression in the brain and intestine of the fish by quantitative polymerase chain reaction (qPCR). The animals were exposed to three salinities: 0, 6, and 12 parts per thousand (ppt) of salt for 21 days. Furthermore, lipid peroxidation, reactive oxygen species, DNA damage, and membrane fluidity in blood cells were quantified by flow cytometry. The results indicated an increased expression of *cart, pyy*, and *cck* and a decreased expression of *npy* in the brain, and the same with *cck* and *npy* in the intestine of fish treated with 12 ppt. This modulation and other adaptive responses may have contributed to the decrease in weight gain, specific growth rate, and final weight. In addition, we showed oxidative damage in blood cells resulting from increasing salinity. These results provide essential data on *O. niloticus* when exposed to high salinities that have never been described before and generate knowledge necessary for developing biotechnologies that may help improve the production of economically important farmed fish.

## 1 Introduction

Nile tilapia (*Oreochromis niloticus*) is a fish species native to African freshwater lakes and rivers. It is currently one of the most widely farmed freshwater fish around the world due to its favorable production characteristics and high economic value and has been used as a biological model in several studies (Beardmore et al., 2001; He et al., 2011; FAO, 2020; Mohamed et al., 2021). Considering the scarcity of fresh water in many countries, studies are being carried out to develop the production of this species in brackish and seawater (El-Sayed et al., 2005; Mohamed et al., 2021).

Changes in water salinity modify the homeostasis of the organisms and biological processes and may even lead to death (Rahmah et al., 2020). Exposure of different tilapia species to increasing salinity is known to affect growth performance (Ninh et al., 2014; Gan et al., 2016), digestive capacity (Tran-Ngoc et al., 2017), blood parameters (Verdegem et al., 1997), reproductive capacity (Cruz Vieira et al., 2019), histopathology and behavior (Hassan et al., 2013), antioxidant status (Gan et al., 2016), and metabolic rate (Iwama et al., 1997).

The homeostatic regulation of food intake depends on a complex network involving signals which promote the release of a wide range of hormones produced by the brain and peripheral organs that can stimulate (orexigenic) or inhibit (anorexigenic) appetite (Conde-Sieira et al., 2018). Among the main hormones involved in appetite regulation, the most important ones are the neuropeptide Y (Npy), cocaine and amphetamine-regulated transcription factor (Cart), cholecystokinin (Cck), and peptide YY (Pyy) (Rønnestad et al., 2017).

Plasma glucose level is one of the most common stress indicators. Furthermore, circulating glucose levels are believed to activate neurocircuitry in regulating food intake (Pankhurst, 2011; Soengas, 2014). In addition, blood oxidative parameters are often used to assess fish health (Bojarski and Witeska, 2020). Thus, evaluating whether salinities caused any damage to tilapia blood cells or whether they modulated plasma glucose levels is extremely interesting.

Living in a marine or freshwater environment, with direct contact with water for gas exchange and secretion of metabolic waste, teleost fish face constant osmotic stress that requires physiological compensation (Mohamed et al., 2021). Under stress conditions, the mechanisms that control food intake in fish are dysregulated. Therefore, understanding the factors underlying the responses to environmental stress observed among species is of great interest, having implications for aquaculture output. Currently, little is known about the influence of salinity on genetic variations related to endocrine factors that regulate appetite in fish, specifically in *O. niloticus*. Therefore, this study aimed to evaluate if exposure to different salinities causes modulation of the expression of genes related to appetite regulation and affects growth rates and the systematic physiology of tilapia.

## 2 Materials and methods

### 2.1 Animals and conditions

The fish used in this study were obtained from the Laboratory of Pisciculture of the Barragem do Chasqueiro Fish Farming (Arroio Grande, Brazil-32°14′15 ″S/53°05′13″ W). The specimens, with initial mean weight and length of 200 ± 8.3 g and 18.5 ± 5.3 cm respectively, were fed twice a day with a commercial diet (Supra, 38% crude protein) until apparent satiety. The fish were kept in plastic tanks with a nominal capacity of 1,000 L filled with 650 L water. The tanks used were opaque to reduce visual stress. Approximately 2/3 of the water in each tank was renewed once a week. The water quality parameters during the acclimation period were as follows: temperature of 24.0 ± 0.34°C; dissolved oxygen level of 9.85 ± 0.1 mg O_2_. L^−1^; salinity of 0 ± 0.21 ppt; pH of 7.04 ± 0.19; and total ammonia level lower than 0.3 mg NH_3_ L^−1^.

### 2.2 Experimental design and sample collection

After 4 weeks of acclimation, fish were randomly distributed into three salinity groups: two experimental groups exposed to 6 and 12 ppt of salt (NaCl) and a control group with fresh water (0 ppt). The salinity levels were obtained by increasing salinity per day until they reached 6 and 12 ppt. The salinity levels mentioned above were maintained throughout the experimental period by regular salt applications, followed by its measurement through a salinometer (Kasvi, Brazil) to ensure the required salinity level daily. The chosen concentrations are within the minimum mortality range (El-Leithy et al., 2019). The experimental groups were exposed for 21 days, and the exposition of all the groups was performed in plastic tanks in triplicate (totaling 9 tanks) with ten animals per tank (*n* = 90 animals) maintained under the same conditions. The water quality parameters were measured daily during the experimental period and were held as follows: Temperature of 24.5 ± 0.40°C; dissolved oxygen level of 11 ± 0.1 mg O_2_. L^−1^; pH of 7.27 ± 0.26; and total ammonia level lower than 0.3 mg NH_3_ L^−1^. At the end of the experiment, all fish were anesthetized in a benzocaine bath (50 mg·L^−1^) and euthanized by cranial spinal section. Their brain and posterior intestine were collected and were used for sequencing, molecular characterization, and gene expression analysis of target genes. The tissues were preserved in liquid nitrogen until their use for molecular biology processing and analyses. Blood samples were collected from the branchial branch artery using 26 G needles attached to heparinized syringes and centrifuged at 2,000×g for 10 min to separate plasma and blood cells (ambient temperature). The plasma osmolality was measured using an automated osmometer (Vapro^®^ Vapor Pressure Osmometer, United States). For flow-cytometry analysis, 20 μl of the blood sample was added to 1 ml of fetal bovine serum (FBS) and stored at 4°C in the dark until use, and the glucose measurement was performed with the Accu-Chek Active Kit (Roche, CH), using 5 μl of blood. The Ethics and Animal Experimentation Committee of Universidade Federal de Pelotas approved the use of animals and all these handling practices (process no 23110.014105/2020-56).

### 2.3 Performance

Fish were individually weighed at the beginning and end of the experimental period. The weight was used to calculate the survival, weight gain, and specific growth rate (Kang’ombe et al., 2008).

Survival (%) = (final number of fish—initial number of fish)/initial number of fish x 100

Weight gain (g) (WG) = FW- IW

Specific growth rate (%/day) (SGR) = 100 (ln FW—ln IW)/Δt.

FW is the final weight, IW is the initial weight, and Δt is the number of days between samplings.

### 2.4 RNA extraction and cDNA synthesis

Total RNA from tissue samples was extracted using TRI Reagent (Sigma, United States), following the manufacturer’s instructions with few modifications. RNA concentration and purity were measured by UV-light spectrophotometry using the NanoVue™ equipment (GE Healthcare Life Sciences, United States), and the samples were then standardized by concentration. Only the samples presenting high purity (OD260/280 ≥ 2.0 nm) were used in the following procedures. According to the manufacturer’s recommendation, RNA samples were treated with DNase using DNA-free™ Kit (Invitrogen™, United States). First-strand cDNA synthesis was performed with 2 µg of total RNA using the High-Capacity cDNA Reverse Transcription Kit (Applied Biosystems, United States) according to the manufacturer’s protocol. The cDNA samples were stored at −20°C until further use.

### 2.5 Target genes in appetite regulation and osmoregulation process

The target genes related to the main hormones that play a critical role in appetite regulation were as follows: Neuropeptide Y (*npy*); Peptide YY (*pyy*); Cocaine and Amphetamine Regulated Transcript (*cart*); Cholecystokinin (*cck*). Genes related to osmotic stress were also analyzed: Sodium-Potassium ATPase (*nka*) and Co-transporter Na^+^/ K^+^/2Cl^–^ (*nkcc*). Their functions are listed in Table 1.

**TABLE 1:** Target genes in appetite regulation, osmoregulation process, and primer sequences used in this study.

Gene symbol	Function	Primer sequence 5′→ 3′	Efficiency (%)	Objective	References
*pyy*	Anorexigenic factor	GGA​CAG​TGC​TGG​TGG​CCT​TAG​TG	—	Cloning	Yan et al. (2017)
		TCT​CTG​GTC​TCT​GTT​GTT​ATC​GCC			
		AAC​ACT​GGC​TGA​TGC​CTA​CC	100.6	qRT-PCR	
		TTC​CAT​ACC​TCT​GCC​TGG​TG			
*cart*	Anorexigenic factor	TGGTCTAYCTGTCCGTCTGTCTG	—	Cloning	
		TAG​CAG​CGC​AGG​AAG​AAG​G			
		TGC​TGA​CAT​CAC​TCT​GTC​AAG​G	98.6	qRT-PCR	
		AGC​CAG​CTC​ACT​GGT​TGT​G			
*cck*	Anorexigenic factor	TCT​CAC​TCT​CAC​ACA​CTC​C	—	Cloning	
		AGG​AGT​ACT​CAT​ACT​CCT​CTG			
		AGA​AAC​TCC​ACG​GCA​AAC​AG	92.5	qRT-PCR	
		ACT​CAT​ACT​CCT​CTG​CAC​TGC			
*npy*	Orexigenic factor	ACA​AGA​CAG​AGG​TAT​GGG​AAG​A	—	qRT-PCR	
		GGCAGCATCACCACATTG			
*nka*	Transport of ions and absorption of water	GCT​CCA​GAG​AGG​ATT​TTG​GAC	—	qRT-PCR	Velan et al. (2011)
		CTC​CAA​GAC​CTC​CCA​ACT​CA			
*nkcc*	Transport of ions and absorption of water	GAG​GCA​AGA​TCA​ACA​GGA​TTG	—	qRT-PCR	Velan et al. (2011)
		AAT​GTC​CGA​AAA​GTC​TAT​CCT​GAA​CT			
*actb*	Reference gene	TGG​TGG​GTA​TGG​GTC​AGA​AAG	—	qRT-PCR	Yang et al. (2013)
		CTG​TTG​GCT​TTG​GGG​TTC​A			

### 2.6 Sequencing and molecular characterization of target genes

The gene fragments were amplified by polymerase chain reaction (PCR) using primers designed by the PriFi online tool (https://services.birc.au.dk/prifi/) after the alignment of known sequences deposited for each gene in GenBank. The PCR parameters were as follows: an initial denaturation step for 1 min at 94°C, followed by 35 cycles at 94°C for 30 s, 55.2–65.5°C (depending on the primer sequence) for 30 s, and 72°C for 1 min, with a final extension of 5 min at 72°C. The fragments were sequenced using the Applied Biosystems 3500 Genetic Analyzer^®^ automatic sequencer (Life Technologies, United States).

### 2.7 Sequence analysis

The partial sequences of the *cart*, *cck*, and *pyy* genes identified in this study were deposited in GenBank. The translation of sequenced nucleotides to amino acid sequences and the open reading frame (ORF) identification were performed using the ExPASy bioinformatics resource portal (https://www.expasy.org/). The conserved domains and sites were mapped using the UniProt database (https://www.uniprot.org/).

### 2.8 Gene expression analysis

Gene expression was analyzed by quantitative polymerase chain reaction (qPCR), and the primers used in this study (Table 1) were designed using the Primer3 online tool (https://primer3.ut.ee/http://bioinfo.ut.ee/primer3–0.4.0/) and the sequences of the identified fragments. The qPCR assay was performed using CFX96 Touch™ Real-Time PCR Detection System (Bio-Rad, United States) and GoTaq^®^ RT-qPCR Master Mix (Promega, United States). The amplification conditions were 95°C for 10 min, 40 cycles at 95°C for 15 s, and 60°C for 60 s, followed by the requirements needed to calculate the melting curve. The 2^–ΔΔCT^ method was used to normalize the fold change in gene expression using *actb* as a reference gene for normalization (Yang et al., 2013).

### 2.9 Flow cytometry analysis

Flow cytometry analyses were performed using Attune^®^ (Acoustic Focusing Flow Cytometer, Applied Biosystems, United States) with violet laser (UV 405 nm-450/40, VL^-1^). To evaluate the effect of different salinities exposure on the systemic physiology of *O. niloticus*, erythrocytes were washed with 500 µl of FBS, stained with Hoechst 33,342 (16.2 mM), and assayed for reactive oxygen species (ROS), lipid peroxidation (LPO), membrane fluidity, and DNA fragmentation index (DFI) (Martinez-Alborcia et al., 2012). Cell debris were discarded by scatter plots of forward scatter × side scatter and negative fluorescence of Hoechst 33,342. To read all parameters, the fluorophore-stained cells were added into calcium- and magnesium-free phosphate-buffered saline (80 g·L^−1^ of NaCl, 11.5 g·L^−1^ of KCl, 24 g·L^−1^ of Na_2_HPO_4_, 2 g·L^−1^ of KH2PO4 dissolved in deionized water). A total of 20,000 cells were measured during each analysis.

#### 2.9.1 ROS evaluation

For the evaluation of ROS produced by the blood cells, 10 µl of previously collected and stored blood sample was added to 20 µl of saline solution containing 2 µM 2′,7′-dichlorofluorescein diacetate, and 5 µM propidium iodide (PI) fluorescent probes (Sigma-Aldrich Co., United States). The samples were analyzed after incubation for 60 min at 22°C in the dark. Only live cells (PI negative) were selected and measured for ROS production by the median intensity of the emitted green fluorescence.

#### 2.9.2 LPO evaluation

The LPO was quantified using 1 µM of the lipid peroxidation sensor 4,4-difluoro-5-(4-phenyl-1,3-butadienyl)-4-bora-3a,4a-diaza-s-indacene-3-undecanoic acid (C11-BODIPY) in 100 µl of the sample. It was incubated for 2 h at room temperature (20°C). The rate of lipoperoxidation was calculated by the median intensity of green fluorescence (peroxidized lipid)/median green fluorescence intensity + median red fluorescence (non-peroxidized lipid) × 100.

#### 2.9.3 Membrane fluidity evaluation

Erythrocyte membrane fluidity was analyzed by hydrophobic merocyanine 540 dye (M540) at a final concentration of 2.7 M (Sigma-Aldrich, United States) and YO-PRO, which fluoresces green, at a final concentration of 0.1 M (Invitrogen, United States). Only live cells (YO- PRO negative) were selected and classified into high fluidity cells (high M540 concentration) and low fluidity cells (low M540 concentration).

#### 2.9.4 DNA damage evaluation

To evaluate DNA damage in erythrocytes, 10 µl of blood sample was added to 5 µl of 0.01 M Tris-HCl, 0.15 M NaCl, and 0.001 M EDTA (pH 7.2) and to 10 µl of Triton 1X (Triton X-100, 1%, v/v) 30 s later. Then, 50 µl of acridine orange dye (2 mg·ml^−1^, #A6014, Sigma-Aldrich, United States) was added to the sample, followed by incubation from 30 s up to 2 min before each reading. The DNA of erythrocytes was classified as undamaged (green fluorescence emission) or damaged (orange/red fluorescence emission). The DNA fragmentation index (DFI) percentage was obtained by the median of red fluorescence intensity/(median of the red + green fluorescence intensities) × 100.

### 2.10 Statistical analysis

The evaluated parameters, which presented normal distribution and homogeneous variance with or without transformations, were analyzed by one-way analysis of variance (ANOVA), followed by Tukey’s post-test with a significance level of 5%. The results from gene expression, ROS, LPO, membrane fluidity, and DFI were expressed as mean ± standard error of the mean (SEM).

## 3 Results

### 3.1 Effects of 21 days of different salinity exposure on nile tilapia growth performance

The final weight, weight gain, and specific growth rate were lower (*p* < 0.05) in the groups exposed to 6 and 12 ppt compared to the control group, as shown in Table 2. Furthermore, the plasma osmolality and the blood glucose showed an increase (*p* < 0.05) in the fish exposed to 12 ppt compared to fish from the control and 6 ppt groups. The survival was significantly lower in the group exposed to 12 ppt compared to the other groups.

**TABLE 2:** Effects of salinity exposure on Nile tilapia growth performance.

	Control	6 ppt	12 ppt
Final weight (g)	295.5 士 11.42^a^	240.8 士 14.69^b^	230.9 士 13.56^b^
Specific growth rate (%/day)	1.70 士 0.38^a^	−0.12 士 0.05^b^	−0.16 士 0.47^b^
Weight gain (g)	72.56 士 12.1^a^	−5.84 士 2.68^b^	−9 士 23.80
Glucose (mg/dl)	60.50 士 9.13^a^	62.17 士 5.41^a^	147.3 士 15.87^b^
Osmolality (mOsm/kg)	325.1 士 4.17^a^	325.3 士 3.70^a^	347.8 士 5.33^b^
Survival (%)	100	100	50

Data are expressed as means ± standard error of the mean. Specific growth rates (%/day) were calculated as SGR = 100 (ln FW—ln IW)/Δt, where IW is the initial weight, FW is the final weight, and Δt is the number of days between samplings. The weight gain (in grams, g) was calculated as WG = final weight—initial weight; and survival (%) = (final number of fish—initial number of fish)/initial number of fish × 100. Different letters indicate significant differences between the experimental groups (one-way analysis of variance; *n* = 30; *p* < 0.05).

### 3.2 Sequencing and molecular characterization

The length of *cart*, *cck*, and *pyy* amplified fragments from *O. niloticus* was 306, 453, and 237 bp, respectively. They were sequenced and deposited under the GenBank accession numbers MW556307.1, MW556308.1, and MW556314.1, respectively.

The partial sequence of *cck* cDNA codes for 136 amino acids on ORF +1. The fragment belongs to the gastrin/cholecystokinin family and has a signal peptide between amino acids 1 and 19 and a gastrin domain between amino acids 4 and 136. The partial sequence of *cart* cDNA codes for 102 amino acids on ORF +1. The fragment belongs to the cart family and has a signal peptide between amino acids 1 and 15. The partial sequence of *pyy* cDNA codes for 79 amino acids on ORF +. The fragment belongs to the NPY family and has a signal peptide between amino acids 1 and 18.

### 3.3 Gene expression analysis

#### 3.3.1 Brain

**FIGURE 1:**
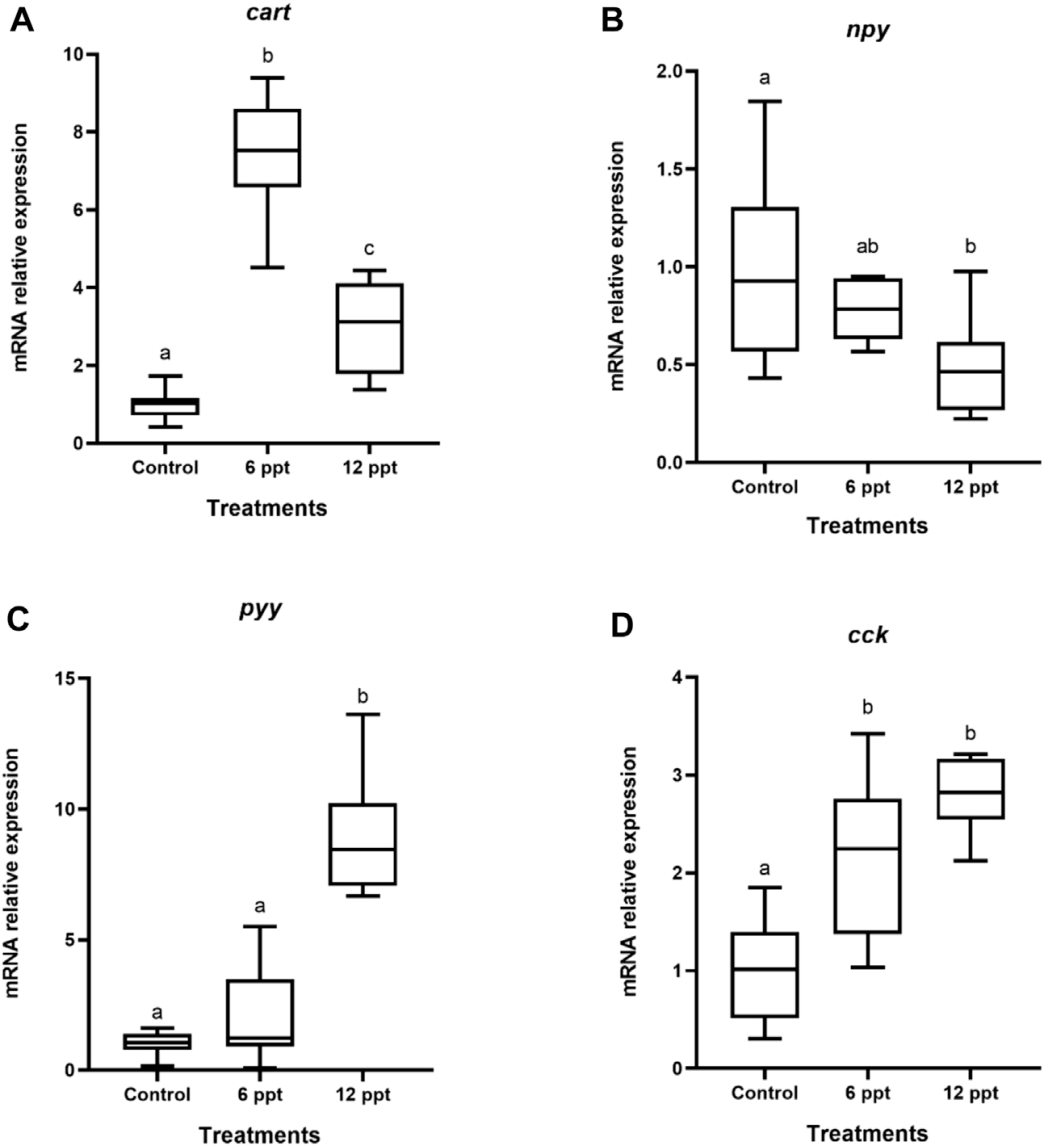
Gene expression in the brain of *Oreochromis niloticus* in the control group and those exposed to 6 and 12 parts per thousand (ppt) of salt for 21 days. The relative expression of the *cart* **(A)**, *npy* **(B)**, *pyy* **(C)**, and *cck* **(D)** mRNA was evaluated by quantitative polymerase chain reaction and normalized using the *actb* gene. The values are expressed as mean ± standard error of the mean. Different letters indicate significant differences between the experimental groups (one-way analysis of variance; *n* = 12; *p* < 0.05)

The mRNA expression of *cart* and *cck* in the brain of the fish exposed to 6 ppt and 12 ppt was higher (*p* < 0.05) than in fish from the control group (Figures 1A,D). The mRNA expression of *npy* in the brain of fish exposed to 12 ppt was lower than in control and 6 ppt groups (Figure 1B). Furthermore, the *pyy* mRNA expression in the brain of fish from the 12 ppt group was higher (*p* < 0.05) than in fish from the other groups (Figure 1C).

#### 3.3.2 Intestine

**FIGURE 2:**
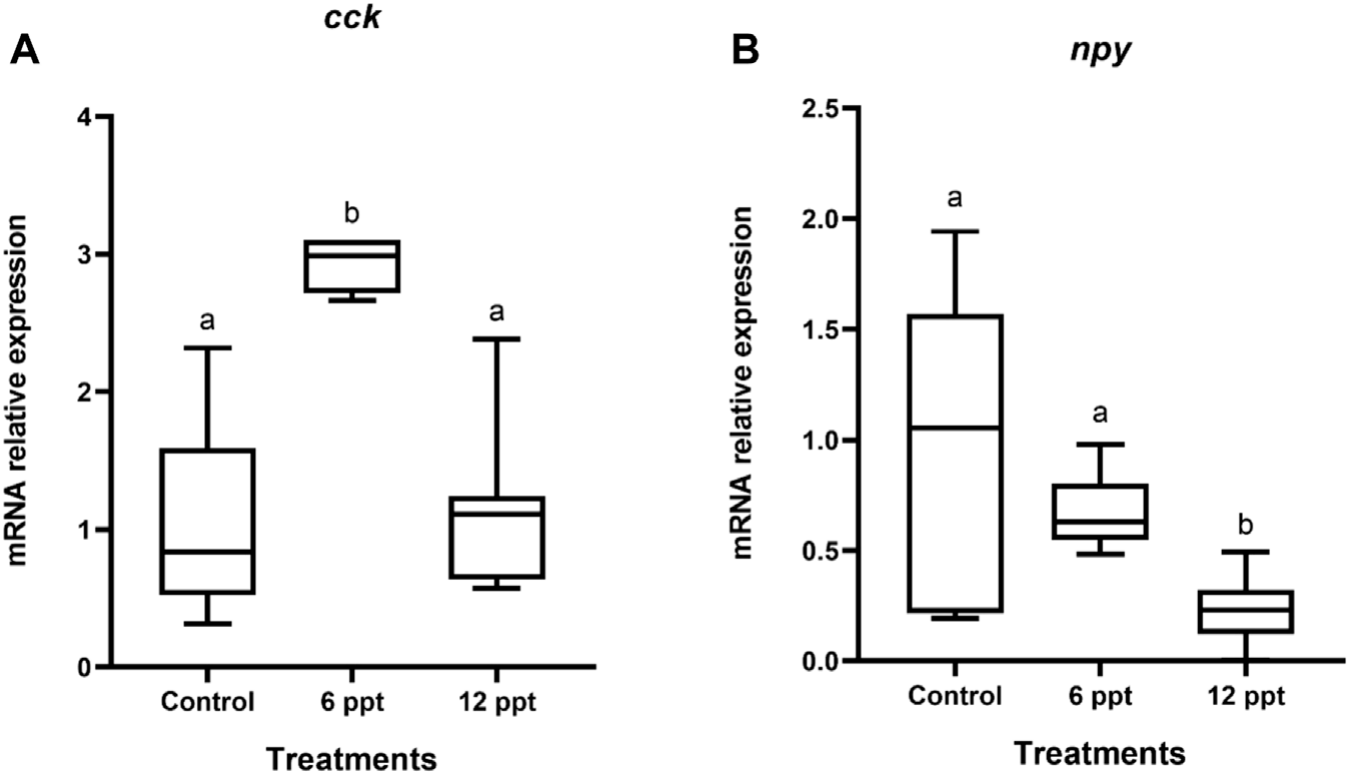
Gene expression in the intestine of *Oreochromis niloticus* in the control group and those exposed to 6 and 12 parts per thousand (ppt) of salt for 21 days. The relative expression of *cck* **(A)** and *npy* **(B)** mRNA was evaluated by quantitative polymerase chain reaction and normalized using the *actb* gene. The values are expressed as mean ± standard error of the mean. Different letters indicate significant differences between the experimental groups (one-way analysis of variance; *n* = 12; *p* < 0.05)

In the intestine, the mRNA expression of *cck* of the fish exposed to 6 ppt was higher (*p* < 0.05) than that in the fish from another group (Figure 2A). The mRNA expression of *npy* in the intestine of the fish exposed to 12 ppt was significantly lower than that in the fish from the control group and those exposed to 6 ppt (Figure 2B).

**FIGURE 3:**
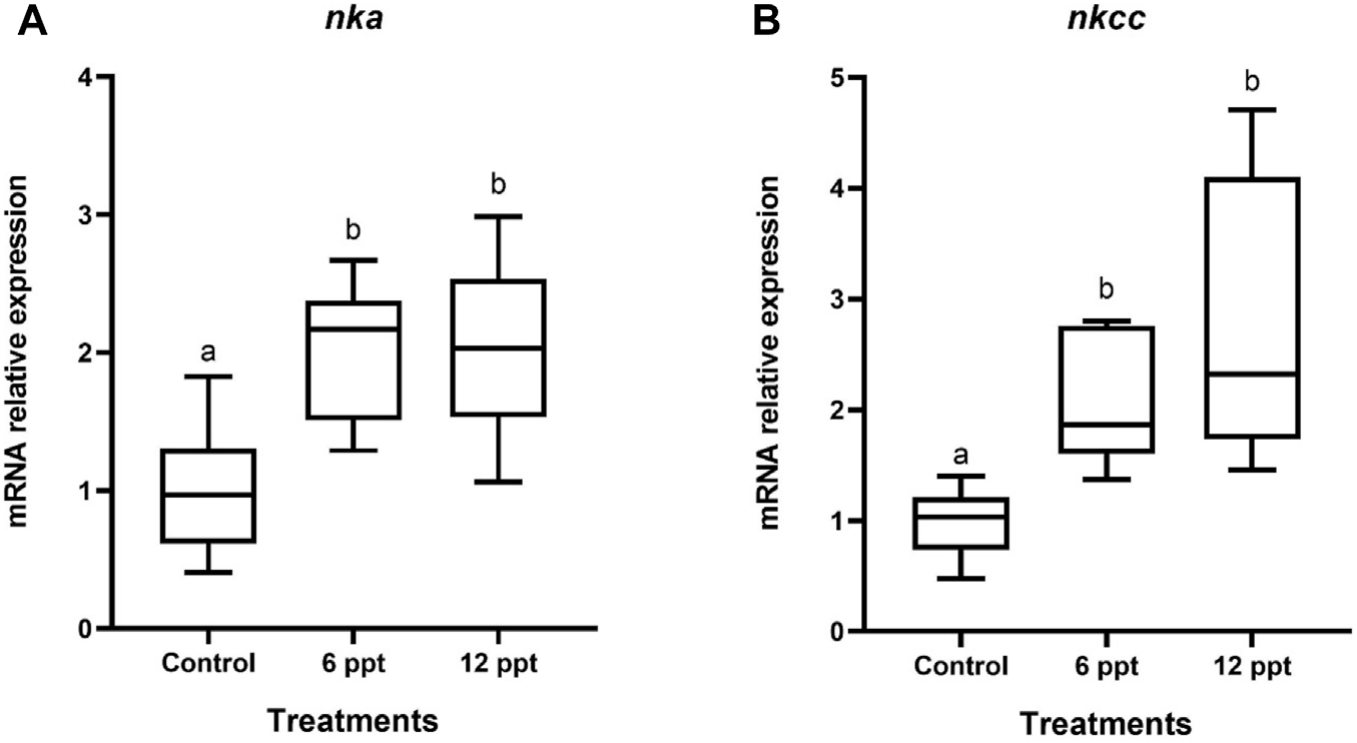
Gene expression in the intestine of *Oreochromis niloticus* in the control group and those exposed to 6 and 12 parts per thousand (ppt) of salt for 21 days. The relative expression of nka **(A)** and nkcc **(B)** mRNA was evaluated by quantitative polymerase chain reaction and normalized using the *actb* gene. The values are expressed as mean ± standard error of the mean. Different letters indicate significant differences between the experimental groups (one-way analysis of variance; *n* = 12; *p* < 0.05).

The mRNA expression of *nka* and *nkcc* in the intestine of the fish exposed to 12 ppt was higher (*p* < 0.05) than that in the fish from another group (Figures 3A,B).

### 3.4 Flow cytometry analysis

**FIGURE 4:**
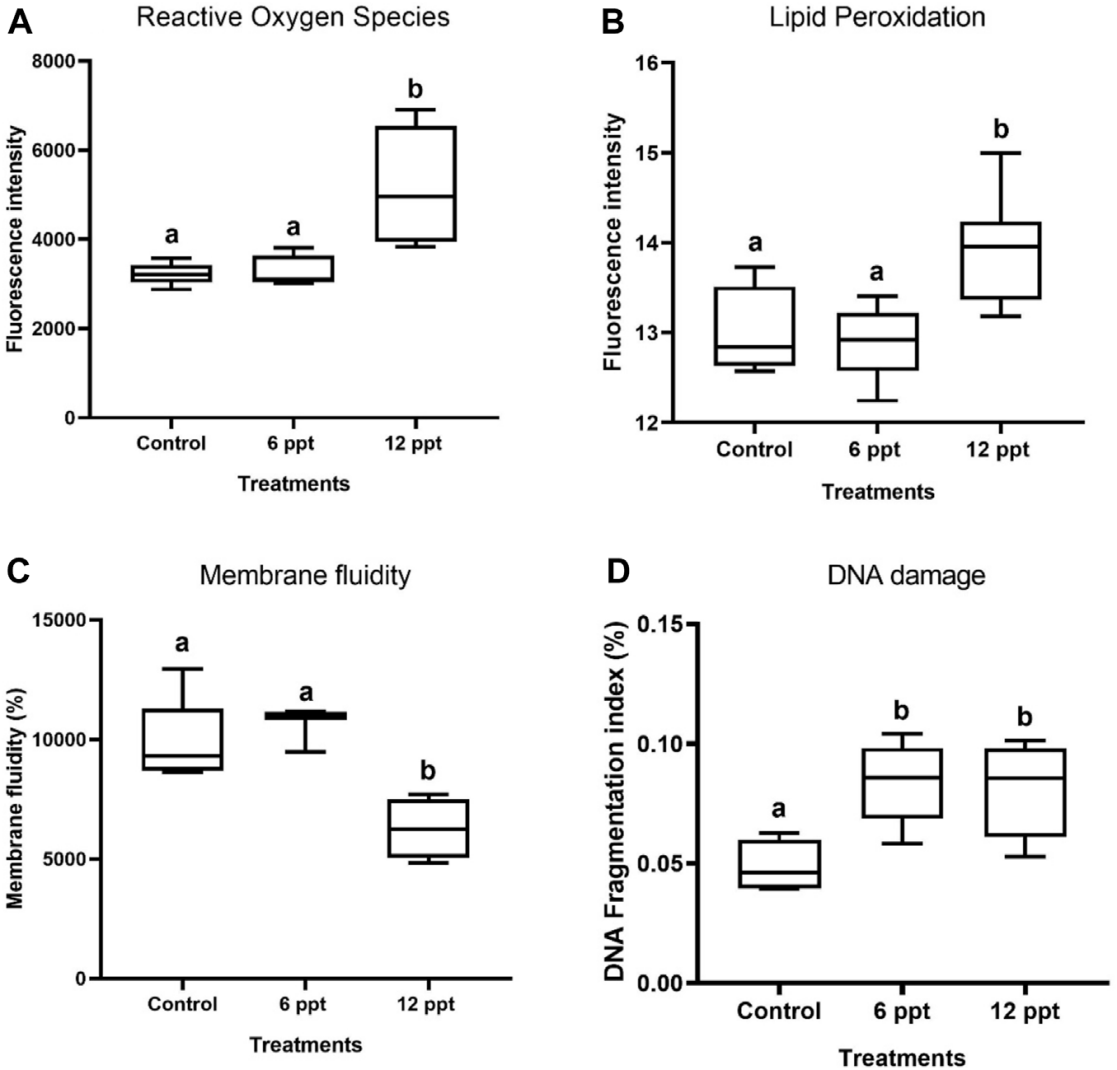
The oxidative effects in terms of reactive oxygen species production **(A)**, lipid peroxidation **(B)**, membrane fluidity **(C)**, and DNA fragmentation index (%) **(D)** in the erythrocytes of Oreochromis niloticus in control and in the groups of fish exposed to 6 and 12 parts per thousand (ppt) of salt, as evaluated by flow cytometry.

The exposure of Nile tilapia for 21 days to12 ppt increased (*p* < 0.05) the ROS (Figure 4A) and LPO levels in erythrocytes (Figure 4B). The membrane fluidity levels in the erythrocytes of fish exposed to 12 ppt showed a significant decrease (Figure 4C). Further, both the concentrations of salinity induced a significant increase (*p* < 0.05) in the DFI of erythrocytes compared with the control group (Figure 4D).

## 4 Discussion

In the present study, the mRNA partial sequences of *cart*, *cck*, and *pyy* in *O. niloticus* were identified and characterized successfully. Furthermore, this was the first study to evaluate the effects of different salinities on the expression of genes related to appetite in Nile tilapia.

The significant decrease in final body weight, specific growth, and weight found in our study is in line with the report of other authors who observed that increased salinity negatively affected *O. niloticus* growth, as well as feed intake and feed conversion ratio in other fish species (Luz et al., 2008; Gan et al., 2016). Researchers suggest that decreased growth in increasing salinity environments is related to reduced food consumption (appetite) (Yan et al., 2004). It is known that feeding in vertebrates is regulated by several factors, including a wide range of orexigenic or anorexigenic hormones, which were analyzed in this study.

Among the anorexigenic hormones, *cart* mRNA is highly expressed in the brain, stomach, and intestine (Ahmadian-Moghadam et al., 2018; Zhang et al., 2018). Here, we observed an increase in the expression of *cart* in the brain, corroborating the report by other authors that this gene may be related to increased appetite. Central administration of Cart inhibits food intake in mice (Kristensen et al., 1998), rats (Stanley et al., 2001) and fish (Volkoff and Peter, 2000).

Npy and Pyy have been extensively studied in several species because of their roles in the mechanism of feeding behavior (Volkoff et al., 2005). Npy injections increase feeding in goldfish (Narnaware and Peter, 2001) and zebrafish (Yokobori et al., 2012). Moreover, Npy treatments have also been shown to stimulate fish growth/growth hormone (GH) secretion both *in vitro* and *in vivo* (Assan et al., 2021). According to what we observed, salinity caused a decrease in the expression of *npy* in the brain and intestine, which could impact appetite modulation in these organs. In turn, Pyy is known to function as an anorexigenic indicator and has been recognized in several fish species, including Atlantic salmon (*Salmo salar*) and goldfish (*Carassius auratus*) (Murashita et al., 2009; Gonzalez and Unniappan, 2010). The increased expression of *pyy* in the brain indicates that this anorexigenic pathway may have been activated by salinity. Cck plays an essential role in food regulation. Here, *cck* had its expression increased by salinity. The literature has already reported that oral Cck administration inhibits feed intake in fish (Rubio et al., 2008). Collectively, these results support the hypothesis that salinity causes modulation in appetite-regulating genes.

Plasma glucose level is one of the most common stress indicators (Pankhurst, 2011; Soengas, 2014). An increase in blood glucose levels in groups exposed to the 12 ppt of salt was observed in the present study. Overall, published data suggest that the fish neural system that controls food intake also responds to stressful situations, increasing plasma glucose and cortisol levels (Conde-Sieira et al., 2018, 2010). It is known that distinct brain regions integrate endocrine and metabolic information to elaborate a coordinated response using neural effector pathways that modulate food intake. These areas contain specialized neurons that use glucose as a signaling molecule. Therefore, glucose-stimulated (GE) neurons increase, while glucose-inhibited (GI) neurons decrease their firing rate as glucose levels increase (Marty et al., 2007). It is already known that neurons in glucose detection areas produce peptides involved in controlling food intake in mammals. Literature data suggest that the arcuate nucleus neurons in the hypothalamus that produce Npy appear to be GI. In contrast, the neurons that produce Cart appear to be GE, resulting in increased *cart* expression and decreased *npy* expression when glucose levels rise, which is in line with the results shown in this study (Dunn-Meynell et al., 2002; Mobbs et al., 2005; Conde-Sieira et al., 2018).

Another standard stress indicator is the blood cortisol level. In fish, one of the responses to stress caused by high salinities involves activation of the hypothalamic-pituitary-interrenal (HPI) axis, which starts with the production of corticotropin-releasing factor (CRF) and culminates in the production of cortisol. Mohamed et al., 2021 observed that blood cortisol levels were increased in fish exposed to higher salinity while investigating the physiological effects of salt stress on Nile tilapia. Previous studies suggest that this responsiveness to stressors is maintained under chronic stress (Gorissen and Flik, 2016) and that higher doses of cortisol profoundly inhibit the feeding behavior of goldfish (Bernier et al., 2004) and sea bass (Leal et al., 2011).

Oxidative parameters in the blood are frequently used to assess fish health (Bojarski and Witeska, 2020). Therefore, to evaluate whether the chosen salinities caused any damage to Nile tilapia blood cells, cell integrity analyzes were performed by flow cytometry. This was the first study that evaluated ROS, lipid peroxidation, membrane fluidity, and DNA fragmentation in blood cells of *O. niloticus* exposed to different salinities using a flow cytometer. It was possible to observe an increase in ROS levels in the higher salinity group. In normal physiological states, there is a balance between pro-oxidant production and antioxidant defenses. When an imbalance in favor of pro-oxidant output occurs, the antioxidant defenses can no longer neutralize the elevated levels of reactive oxygen species. Studies have already shown that salt stress induces the overproduction of ROS, which can disrupt metabolism and weaken the immune system, eventually leading fish to death (Liu et al., 2007). It is known that an imbalance in ROS and antioxidative enzymes production, leading to a bigger concentration of ROS, can increase lipid peroxidation, and this was observed in the present study. Our result corroborates with other authors who reported the increase in membrane damage caused by salinity (Sinha et al., 2014; Gan et al., 2016; Mohamed et al., 2021).

The change in membrane fluidity observed in the present study may have occurred because cell membranes are susceptible to disturbance from salinity changes. Sodium interacts with membrane lipid components, and increases in the concentration of sodium ions near the membrane lipids can alter membrane geometry and modulate the interaction with other macromolecules nearby (Evans and Kültz, 2020). We also observed an increase in the rate of genomic DNA damage, which could have been due to the increased ROS in the blood, or as a direct response to salinity exposure. DNA is a cellular macromolecule whose structure and function can be affected by changes in intra and extracellular ion concentrations that accompany salt stress (Fiol and Kültz, 2007). Due to its negative charge, DNA becomes susceptible to interaction with cations, such as Na^+^, which can increase or decrease in abundance after the change in salinity. However, deviation from the optimal concentration of intracellular cations can result in irregular interactions with nucleic acids and changes in DNA structure and function (Várnai and Zakrzewska, 2004). Consistent with the destabilizing effect of sodium on nucleic acids, high levels of sodium cause DNA strand breaks in various organisms (Evans and Kültz, 2020). Previous studies demonstrated that fish positively regulate transcripts and proteins indicative of DNA damage as part of the salinity stress response. The presence of widespread strand breaks in marine organisms results from living in high sodium environments. These DNA strand breaks persist until salinity is reduced, emphasizing hyperosmotic stress’s harmful effects (Dmitrieva et al., 2006; Whitehead et al., 2013).

The intestine displays important functions for absorbing nutrients and, as a barrier to the external environment, it has the additional function of maintaining the necessary absorption of the active fluid in seawater (Grosell, 2010). Increased active fluid absorption in seawater is associated with increased Na^+^, K^+^-ATPase (NKA) activity throughout the intestinal canal. The co-transporter Na^+^/K^+^/2Cl^–^ (NKCC) together with NKA, contributes to the transport of ions and absorption of water. Here, an increase in the expression of *nka* and *nkcc* genes was observed, showing that the fish were under osmotic stress. These results agree with Mohamed et al. (2021), who observed a significant increase in the gene expression of *nka* in *O. niloticus* in groups with salt stress after 10 days of exposure. Similar results were also reported in rainbow trout (*Oncorhynchus mykiss*) and Mozambique tilapia. (Kammerer et al., 2009; Kammerer and Kültz, 2009; Gilmour et al., 2012).

From the analysis performed, it was possible to observe an increase in glucose and oxidative damage in *O. niloticus* blood cells exposed to 12 ppt. Furthermore, after 21 days, we observed modulation in the expression of *cart, npy, pyy, cck, nka,* and *nkcc*. These adaptive salinity responses may have helped to decrease appetite in tilapia since the anorexigenic genes were upregulated while the orexigenic factors were deregulated. Consequently, this modulation may have contributed to the decrease in weight gain, specific growth rate, and final weight.

The first death was observed in the middle of the experimental period. At the end of 21 days, we observed 50% of mortality in 12 ppt group, probably due to the harmful effects on Nile tilapia physiology, reinforcing the damage caused by salinity in this species. It also provides essential data on impact of salinity on Nile tilapia’s physiology, which has never been described before. More comprehensive studies are still needed to better understand the effects of salinity on gene expression in Nile tilapia. However, the knowledge generated in this study can serve as a basis for selecting individuals or for the genetic improvement of Nile tilapia through genetic engineering.

## Data Availability

The datasets presented in this study can be found in online repositories. The names of the repository/repositories and accession number(s) can be found in the article/supplementary material.
